# Sustainable nutrition: integrating food systems, environmental science, and diet to advance public and planetary health

**DOI:** 10.3389/fnut.2026.1976376

**Published:** 2026-10-26

**Authors:** Andrew A. Bremer, Anna Krans, Daniel J. Raiten

**Affiliations:** Office of Nutrition Research, National Institutes of Health, Bethesda, MD, United States

**Keywords:** nutrition ecology, nutrition science, planetary health, public health, systems science

## Abstract

Nutrition science has traditionally focused on dietary intake, nutrient metabolism, physiology, and health outcomes. Although these domains remain fundamental, advancing public health increasingly requires understanding nutrition within the broader systems that shape food production, processing, access, consumption, and environmental sustainability. This Perspective proposes sustainable nutrition as a unifying concept: maintaining nutritional status that supports growth, development, and health across the lifespan while accounting for population needs, context, and the capacity and resilience of food systems. We describe a Nutritional Ecology framework that conceptualizes nutrition as the product of dynamic, reciprocal interactions among human biology, dietary patterns, food systems, and environmental conditions. Agricultural practices affect food quantity, quality, nutrient composition, biodiversity, water resources, greenhouse gas emissions, and ecosystem resilience, while environmental change influences crop productivity, nutrient density, food safety, and nutrition security. Dietary patterns simultaneously affect chronic disease risk and the environmental footprint of food systems. These interdependencies argue against evaluating human health and environmental sustainability in isolation. We therefore call for a transformation toward integrated, systems-based nutrition research that incorporates biological variability, environmental drivers, and system-level interactions. Multidisciplinary collaborations spanning nutrition, food, agricultural, environmental, epidemiologic, computational, systems, and implementation sciences will be essential. Emerging approaches, including systems mapping, network analysis, computational modeling, artificial intelligence, and multiscale data integration, can identify trade-offs and leverage points for intervention. Integrating nutrition, food systems, and environmental science is a scientific necessity for generating evidence across diverse population contexts that supports resilient food systems, effective dietary guidance, improved clinical care, healthier people, and a healthier planet.

A fundamental goal of nutrition research is to understand how diet and nutritional status influence health across the lifespan and to translate that knowledge into effective interventions, clinical practice, and public policy. In addition to the ongoing efforts by the nutrition research community to advance our understanding of the intimate role of nutrition in all aspects of health promotion and disease prevention, enhanced efforts to define nutrient requirements or identify optimal dietary patterns need to integrate an increased emphasis on rigorous, transparent approaches that account for biological variability, context, and the accuracy and reliability of measurement. This approach will necessitate a broader view of nutrition – one that recognizes a complex set of interactions between the various “environments” that comprise the human nutritional ecology including the systems in which food is produced, processed, accessed, and consumed (1–3) (Figure 1). Acknowledging the importance of this ecology to food production and consumer choices, and ultimately human and planetary health, is key to the development of etiologically based, context-specific interventions and practices that are both effective and avoid unintended consequences.

**Figure 1:**
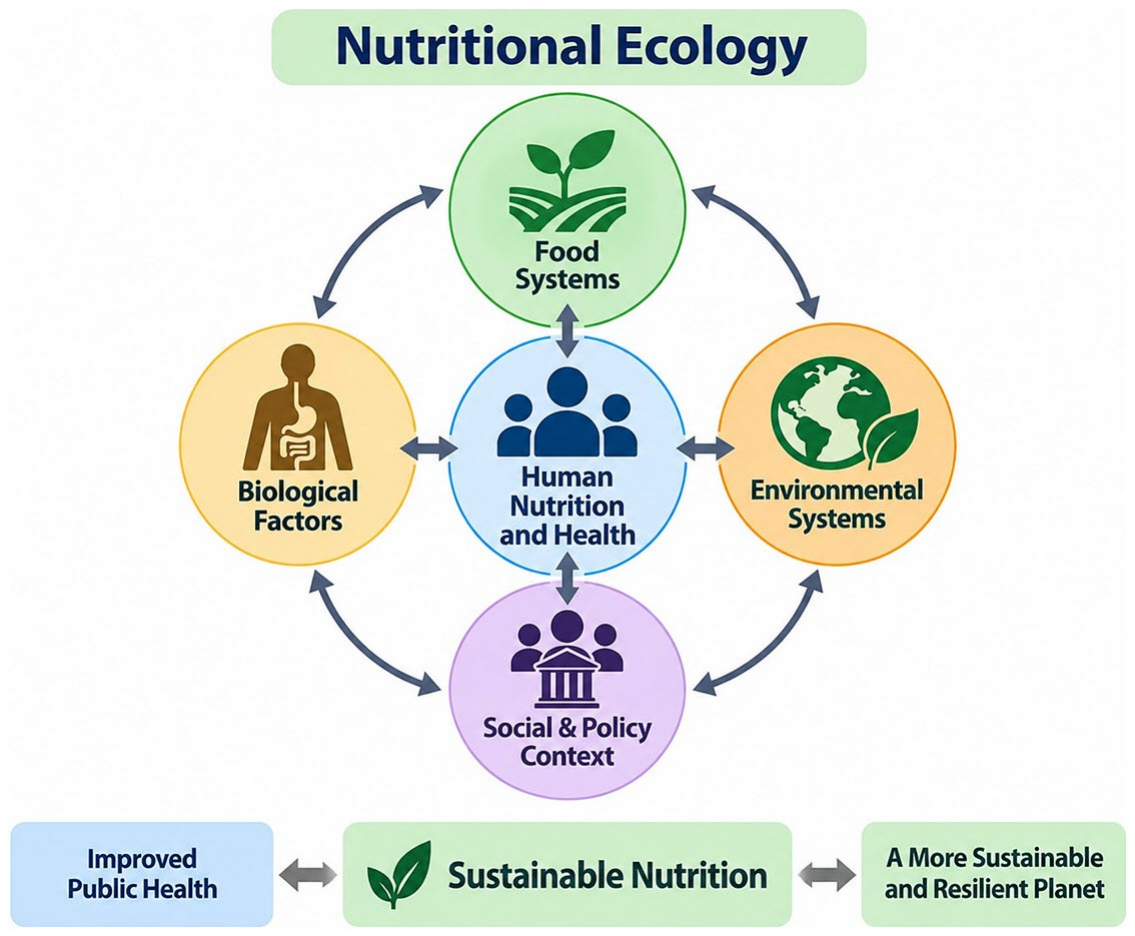
A schematic representation of the nutritional ecology in the context of sustainable nutrition.

This more integrated framework inspires a new conceptual approach to achieve not just dietary sufficiency but sustainable nutrition, i.e., maintaining a nutritional status that supports growth, development, and health across the lifespan while recognizing the needs and context of populations and the capacity of food systems to meet those needs.

Looking forward, the goal needs to shift to improving the public health of the growing global population while optimizing the environmental systems on which food production depends (4–9). This challenge extends beyond food production to the dynamic interactions between agriculture, food processing, dietary patterns, environmental change, and human biology. Addressing these interactions requires a unifying framework that brings together agricultural, food, nutrition, environmental, policy, and health sciences (4–9). Agricultural systems influence food availability, nutrient quality, biodiversity, water resources, greenhouse gas emissions, and ecosystem resilience. Agricultural practices are also synergistically impacted by environmental changes; these, in turn, impact crop productivity, nutrient density, food safety, and dietary choice and exposures (4–9). Concurrently, dietary patterns influence production choices and ultimately both chronic disease risk and the environmental footprint of food systems (4–9). These reciprocal relationships demonstrate that food and agricultural systems, environmental health, and nutrition are inseparable.

To support the evolution of our understanding of this complex set of interactions, we propose an integrated, systems-based framework that links nutrition and health with the sustainability of food systems. We further suggest that this framework be accompanied by a research agenda that engages multidisciplinary teams capable of addressing the complex interactions among human biology, food systems, and environmental conditions. Sustainable nutrition cannot be fully understood – or effectively advanced – through nutrition science alone. It requires an integrated scientific framework linking dietary patterns, food/agricultural systems, environmental science, and public policy interventions (4, 5, 7).

Critically, nutrition science has and must continue to focus on dietary intake, nutrient metabolism, physiology, and health outcomes (3, 10). These domains remain fundamental and have provided the scientific foundation for dietary guidance and clinical nutrition. We are suggesting human nutrition is shaped by broader physical, social, economic, cultural, and environmental contexts which influence food production, food access, dietary choices, and health – including food affordability, market dynamics, and the real-world adoption of emerging products. Thus, understanding nutrition requires expanding beyond historical paradigms toward more integrated systems-based models capable of integrating environmental and biological complexity.

The concept of a “Nutritional Ecology” provides such a framework by recognizing nutrition as the product of dynamic interactions among food systems, environmental conditions, biological processes, and health across the lifespan (1–3). Within this framework, nutritional status operationally serves as an indicator of how well diet and biology are working to perform the functional role of dietary constituents in functional systems to support health and development. Nutritional status functions both as an input and an outcome of interactions among biological processes, food systems, and environmental conditions. Agricultural practices influence not only food quantity but also food quality and nutrient composition. Food processing shapes dietary patterns and exposure to additives and contaminants. Climate conditions and environmental change alter crop nutrient density, food safety, and nutrition security, while dietary choices influence agricultural production and environmental sustainability (4–9, 11–15). Nutrition, therefore, cannot be evaluated independently of the systems that produce food or the environments in which people live and make choices about their health (16).

These interdependencies have profound implications for both planetary and public health. Soil degradation, biodiversity loss, and extreme weather increasingly threaten food production and the nutritional quality of diets (13–16). Simultaneously, modern food systems characterized by resource-intensive production and widespread consumption of ultra-processed foods contribute to obesity, cardiometabolic disease, and environmental degradation (11, 12, 17). These reciprocal relationships underscore the concept of sustainable nutrition (16). This integrated perspective is also consistent with the broader ‘One Health’ paradigm, which recognizes the interdependence of human, animal, plant, and environmental health and increasingly emphasizes systems-based approaches capable of identifying co-benefits and trade-offs across these domains (18, 19). Both of these frameworks recognize that addressing either human health or environmental sustainability in isolation risks generating incomplete evidence and ineffective interventions. Sustainable nutrition, therefore, requires balancing the biological needs of individuals and populations with the environmental resilience and productive capacity of food systems. Human health and environmental sustainability are not competing priorities; they are fundamentally interconnected (16, 20–23).

Achieving this vision of sustainable nutrition requires a corresponding transformation in nutrition research. Traditional linear, single-discipline approaches are insufficient for evaluating systems in which agriculture, environmental conditions, food processing, dietary behavior, and human biology interact continuously. Analyses that focus narrowly on food supply, diet, or health without considering biological variability, environmental drivers, and system-level interactions risk producing incomplete evidence and unintended consequences. Integrated frameworks can instead identify trade-offs and inform context-specific, resilient strategies that advance nutritional adequacy, environmental sustainability, and long-term health (4–9). As such, future research should intentionally integrate nutrition scientists with agricultural scientists, environmental scientists, food scientists, epidemiologists, computational scientists, systems modelers, and implementation researchers (24, 25). Such collaborations are essential for generating evidence that simultaneously optimizes nutritional adequacy, environmental sustainability, and population health.

Progress will require intentional collaboration across disciplines. Nutrition scientists can define biological and health outcomes; food scientists can address processing, safety, and formulation; agricultural scientists can evaluate production and sustainability; and environmental scientists can characterize the ecological conditions that shape food systems and health. Computational approaches – including systems mapping, network analysis, modeling, and multiscale data integration – can help characterize, analyze, visualize, and predict these interactions and identify leverage points for intervention. The importance of economists and agroeconomists must also not be overlooked, as well as assessments of the nutritional life cycle (26).

Fortunately, the scientific tools needed for this transition are rapidly emerging. Advances in systems science, computational modeling, network analysis, artificial intelligence, and multi-scale data integration now permit investigation of complex biological and environmental interactions previously difficult to quantify. These approaches provide opportunities not only to better understand nutrition but also to evaluate trade-offs among dietary quality, environmental sustainability, agricultural productivity, and health outcomes. Moving forward, nutrition science must account for the complex biological, food, social, and environmental systems that sustain, constrain, and direct human nutrition. The Office of Nutrition Research at the National Institutes of Health is poised to support these efforts and advance priority research agendas that embrace an integrated, systems-based approach to nutrition, food systems, environmental science, and health outcomes.

This comprehensive, integrated approach is exemplified by a recently published series of reports under the aegis of the Agriculture and Diet: Value Added for Nutrition, Translation, and Adaptation in a Global Ecology (ADVANTAGE) project (16). The project’s core premise is that developing evidence-based programs, guidelines, and interventions requires analyzing data across three pillars: (i) what is known about priority diet and health relationships; (ii) what people eat and why (i.e., the intersection of dietary patterns and the food environment); and (iii) the capacity of food systems to provide the food and nutrients needed to meet established dietary goals. Building on this framework, the ADVANTAGE project examined whether and how a changing environment affects these pillars, and how such interactions can be integrated into future analytical frameworks to support the translation, development, and implementation of evidence-based programs and guidance. These analyses were presented in a series of manuscripts intended to: (i) stimulate a targeted research agenda; and (ii) inform the efforts of agencies and organizations engaged in developing and promulgating evidence-based approaches to diet and health in the United States and globally (16).

Ultimately, advancing public and planetary health requires rethinking how nutrition research is conceptualized. Nutrition research should not be limited to the study of nutrients and metabolism, as important as that endeavor remains, but should be expanded to include the study of dynamic biological systems embedded within equally dynamic food and environmental systems. Integrating food systems, environmental science, and nutrition is therefore not simply an opportunity – it is a scientific necessity. Such integrated research approaches may also contribute to progress across multiple United Nations Sustainable Development Goals by addressing the interdependent challenges of nutrition, health, food systems, and environmental sustainability. By embracing this systems-based framework, investigators can generate new hypotheses and ultimately the evidence needed to build resilient food systems, strengthen dietary guidance, improve clinical care, and ensure that nutrition science contributes simultaneously to healthier people and a healthier planet.

## Data Availability

The original contributions presented in the study are included in the article/supplementary material, further inquiries can be directed to the corresponding author.
